# Curative Properties of Oil of Turpentine

**Published:** 1854-03

**Authors:** C. A. Hathwell

**Affiliations:** Virginia, Cass Co., Ill.


THE NORTH-WESTERN

MEDICAL AND SURGICAL JOURNAL.

NEW SERIES.

VOL. III. MARCH, 1854. NO. 3.

### ORIGINAL COMMUNICATIONS.

ART. I.—*Curative Properties of Oil of Turpentine.* By C. A. HATHWELL, M. D., of Virginia, Cass Co., Ill.

I HAD prepared a paper some time since for publication in this Journal, on the importance of the Oil of Turpentine in the treatment of various diseases, but in consequence of professional and other engagements, it laid "like a warrior taking his rest" quiet and undisturbed in my office, till my attention was again directed to it by reading the remarks of Dr. Thompson in the No. for December, 1853.

I fully coincide in the opinion of that gentleman, that it is an article too much neglected by the profession generally. When a medical student in Philadelphia, some twenty years since, Professors Coxe and Chapman both appeared much opposed to it, and were very chary in its recommendation. Judging from their high authority, I embarked in practice with early prejudices imbibed against it, but as time rolled on, and I began to do my own thinking and ceased looking through the spectacles of others, I used the article extensively, and am now satisfied it is a valuable and important remedy in ameliorating "the ills which flesh is heir to."

If worthy of record in these pages, I would submit a few cases in which I have employed it with the most unequivocal advantage.

February 10, 1850. Called to Mrs. W. S., on the Indian Creek settlement, found her laboring under the following symptoms :—

pulse small, hard, and corded, abdomen tense, complained of great pain on pressure, particularly about the umbilicus, skin dry, much gastro-intestinal irritation, difficulty in voiding urine, restlessness, anxiety, intense thirst, tongue furred, breath hot and offensive, etc. She stated she was taken with a chill, afterwards pyrexia.

I diagnoseed it a case of enteritis, bled my patient freely, ordered hops stewed in vinegar to be kept constantly to the bowels and hyd. proto. chlo. gr. viij. div. in pulv. No. 8, one to be given every hour and followed with a full dose of oil.

Feb. 11th. Patient not decidedly relieved, applied large blister over abdomen, directed emollient enemata to be thrown up the bowels in large quantities, mucilaginous drinks, etc.

Feb. 12th. Patient improved, ordered castor oil and turpentine; great objections made to the latter article, with the observation, "Why, Doctor, we use that in paint and to kill chintzes" the notable old nurse with uplifted hands and eyes dilated, disapproved of the prescription, but nevertheless under this treatment there was a gradual and general subsidence of pain, pulse became fuller and softer, moderate diaphoresis, skin soft, fecal evacuations natural, etc., and my patient rapidly convalesced.

June 4, 1850. I visited a son of Mr. P. E. R., aged 15, affected with dysentery, saw him several days. I employed the usual remedies without any decided benefit, one day better, and then again relapsing into the same state, or worse perhaps than when first called to see him. I finally concluded on resorting to the Ol. Ricini et Terebinth., and in a short time the lad had so much recovered, that my visits were unnecessary.

July 1, 1852. Visited Mrs. P., laboring under dysentery of a chronic character. The case was treated principally with the oil and turpentine, and she soon recovered.

I can observe, that in my hands I have found no agent so positively beneficial in either the acute or chronic forms of dysentery, or in any other diseased action of the mucous membranes.

For some time past I have been in the habit of prescribing the turpentine and oil in the convulsive affections of children, which are mostly attributed to worms, and with evident advantage. I now rarely employ any of the anthelmintics that are commonly recommended, but from experience place my reliance upon these two articles in combination. Their modus operandi seems to consist in relieving the stomach and intestines from any irritating matter that may have accumulated in them, and then also lubricating and healing the irritated membranes.

During the month of August, 1853, several cases of Cholera Infantum came under my management, one of which I relate, as it, with little variation, was characteristic of nearly all.

August 8. Was sent for to see a child of Mr. J. B., a fine little boy 22 months old, had almost constant watery discharges from the bowels, extremely fetid and unpleasant, stomach excessively irritable, everything instantly ejected, pulse quick, cold extremities, etc. ℞ Sub. Mur. Hyd. gr. l. c. Sacch. Alb. ℈j Pulv. Cinnam. ʒss. M. et. div. in chart. No. 10, one to be administered every hour, and a mild sinapism over the cardiac region.

Next day, found the stomach yet quite irritable, head hot, thirst great, colliquative stools, etc., ordered cloths wrung out of cold water to the head, demulcents, as flaxseed tea for drink, and tapioca, continued the calomel.

Aug. 10. Found the irritability of the stomach had abated, but the alvine evacuations very frequent. I directed small doses of the Oleag. mist. cum creta.

Aug. 11. Observed the abdomen much swollen, tenderness on pressure, symptoms of cerebral oppression, dejections of a sanious character and slimy. I commenced the use of oil and turpentine.

Aug. 13. Two days after exhibiting the above remedy, the tumefaction of the abdomen had entirely subsided, the fecal discharges assumed a more natural appearance, and the improvement general. In a few days I had the proud and joyful satisfaction o feeling and beholding that under Providence, I had been the humble instrument of rescuing that, darling child from the very jaws of death, and restoring it to the embraces of its doting parents, a pleasure that none are more competent to appreciate than the practising physician, and one especially whose peaceful mansion the insatiate archer has invaded, and stricken down its jewel.

The task would not be difficult to record some other cases in which I have employed the turpentine with marked advantage as in certain hemorrhages, etc., but as brevity is desirable in all communications, I shall forbear, having no disposition to intrude further upon the space allotted to a correspondent, to the exclusion perhaps of more important matter.

In conclusion, I must observe, that I do not recommend the turpentine as a panacea, as some physicians, I regret to say, will attach their names to a popular nostrum, and give it notoriety, but as a valuable therapeutic agent considerably lost sight of.

